# Antibody-Drug Conjugates and Their Potential in the Treatment of Patients with Biliary Tract Cancer

**DOI:** 10.3390/cancers16193345

**Published:** 2024-09-30

**Authors:** Shaun Alexander, Umair Aleem, Timothy Jacobs, Melissa Frizziero, Victoria Foy, Richard A. Hubner, Mairéad G. McNamara

**Affiliations:** 1 Department of Medical Oncology, The Christie NHS Foundation Trust, Manchester M20 4BX, UK; umair.aleem@hse.ie (U.A.); melissa.frizziero@nhs.net (M.F.); victoria.foy@nhs.net (V.F.); richard.hubner@nhs.net (R.A.H.); 2 The Library, The Christie NHS Foundation Trust, Manchester M20 4BX, UK; timothy.jacobs@nhs.net; 3 Division of Cancer Sciences, School of Medical Sciences, University of Manchester, The Christie NHS Foundation Trust, Manchester M20 4BX, UK

**Keywords:** antibody–drug conjugate, biliary tract cancer, anti-HER2, payload, targeted therapy

## Abstract

**Simple Summary:**

Survival for patients with biliary tract cancer (BTC) is poor, especially in the advanced stage, where curative treatments are not available. While chemotherapy and immunotherapy are used in standard practice, antibody–drug conjugates (ADCs) have emerged as a novel therapy option. ADCs potentially enhance cancer cell death and reduce side effects. The anti-HER2 ADC Trastuzumab Deruxtecan (T-Dxd) has shown survival benefit for patients whose tumours are HER2-positive, including a subgroup of BTCs. In April 2024, the regulatory authority FDA approved T-Dxd for adults with unresectable or metastatic HER2-positive solid tumours, including BTCs, lacking other treatment options. Ongoing trials are exploring other potentially actionable BTC molecular alterations. This review will discuss the current evidence and ongoing research in this subject area.

**Abstract:**

**Background:** Biliary tract cancers (BTCs) are aggressive in nature, often presenting asymptomatically until they are diagnosed at an advanced stage. Surgical resection or liver transplantation are potential curative options. However, a large proportion of patients present with incurable locally advanced or metastatic disease and most of these patients are only eligible for palliative chemotherapy or best supportive care. More recently, targeted therapies have proven beneficial in a molecularly selected subgroup of patients with cholangiocarcinoma who have progressed on previous lines of systemic treatment. However, only a minority of patients with BTCs whose tumours harbour specific molecular alterations can access these therapies. **Methods:** In relation to ADCs, studies regarding use of antibody–drug conjugates in cancer, particularly in BTCs, were searched in Embase (1974 to 2024) and Ovid MEDLINE(R) (1946 to 2024) to obtain relevant articles. Examples of current clinical trials utilising ADC treatment in BTCs were extracted from the ClinicalTrials.gov trial registry. **Conclusions:** Overall, this review has highlighted that ADCs have shown encouraging outcomes in cancer therapy, and this should lead to further research including in BTCs, where treatment options are often limited. The promising results observed with ADCs in various cancers underscore their potential as a transformative approach in oncology, warranting continued exploration and development and the need for education on the management of their specific toxicities. By addressing current challenges and optimising ADC design and application, future studies could potentially improve treatment outcomes for patients with BTCs and beyond, potentially in both early and advanced stage settings.

## 1. Introduction

Biliary tract cancers (BTCs) are a group of malignancies associated with high morbidity and mortality rates. Although these cancers are rare, making up approximately 3% of all gastrointestinal neoplasms, global data highlights a consistently rising incidence over the past 30 years [1]. BTCs encompass a diverse and challenging group of malignancies, including cholangiocarcinoma (CCA), gallbladder cancer (GBC), and ampullary cancer. CCAs can be further categorised based on their anatomical location into intrahepatic (iCCA) and extrahepatic (eCCA) types. Extrahepatic cholangiocarcinoma (eCCA) itself includes perihilar (pCCA) and distal (dCCA) cholangiocarcinoma [2]. BTCs are known for their rarity and aggressiveness, resulting in poor prognostic outcomes, making them notoriously difficult to treat. The global incidence of BTC varies across geographical areas, with lower rates observed in Western Europe (2.00–3.59 cases per 100,000 annually) and North America (2.33–2.35 cases per 100,000 annually) [3]. In contrast, the Asia–Pacific region and South America exhibit higher incidence rates, likely due to a higher prevalence of specific risk factors such as chronic liver disease, liver fluke infections, obesity, and excessive use of alcohol and tobacco [3]. BTC outcomes remain poor, as demonstrated by a retrospective analysis of 13,827 patients with metastatic BTC over 17 years, revealing a median overall survival (OS) of just 4.5 months [4]. In another large cohort of Korean patients with localised BTCs, the 5-year survival rate was 48.5% for ampullary cancer, 28.5% for GBC, 19.9% for eCCA, and 10.8% for iCCA [5]. 

Surgical resection remains the mainstay of curative treatment for patients with BTCs; however, only 20% of patients have disease amenable to surgery [6]. Moreover, surgery is a high-risk procedure, contraindicated in some patients due to co-morbidities or increasing age. Adjuvant chemotherapy with capecitabine is now an approved standard of care option in patients with radically resected CCA or GBC, following the results of the phase III randomised BILCAP study [6]. However, relapse rates remain high, with 60% at 5 years [7]. The vast majority of patients with BTCs present with a locally advanced or metastatic stage disease, and for these patients, palliative chemotherapy or best supportive care are often the only feasible treatment options. More recently, molecularly targeted therapies including, but not limited to, the inhibitors of the isocitrate dehydrogenase 1 (IDH1) and fibroblast growth factor receptor (FGFR) have proven effective in a molecularly selected subgroup of patients with iCCA after at least one previous line of systemic treatment and have received approval by international regulatory agencies for use in this setting [8,9,10,11]. However, only a minority of patients with BTCs (approximately 15% of those with iCCA) exhibit relevant molecular alterations (i.e., *IDH1* mutation, *FGFR2* fusion or rearrangement), making them eligible for these treatments [12,13,14]. Other molecular alterations have also been identified such as v-raf murine sarcoma viral oncogene homolog B (*BRAF*) and human epidermal growth factor 2 (*HER2*), with research ongoing [2]. 

Increasingly ageing populations remain an issue, particularly in developed nations [15]. Chronic diseases associated with ageing, such as cancer, cardiovascular and cerebrovascular diseases, neurodegenerative disorders, and metabolic conditions, have become major health concerns in these regions [15,16]. The medical costs for treating these conditions are taking up a growing share of government and insurance budgets. As a result, drug discovery and development in the coming decades are expected to focus primarily on addressing these chronic diseases [17,18]. Novel anti-cancer targeted therapies, such as antibody–drug conjugates (ADCs), are increasingly being researched and utilised, aiming to offer effective alternatives, especially in patients where treatment options are often limited to chemotherapy or immunotherapy. 

This review will outline the structure and mechanism of ADCs and highlight their application in various cancer types, including lung, breast, and urothelial cancers, to showcase their current uses and efficacy. Furthermore, the review will explore how these insights could be extrapolated for the treatment of BTCs. The data on the efficacy and safety of ADCs in BTC is currently limited, primarily due to the rarity and complexity of the disease. BTC is a diverse group of cancers, including cholangiocarcinoma, gallbladder cancer, and ampullary cancer, with distinct molecular characteristics. As a result, effective targeted therapies, including ADCs, are still in the early stages of exploration for this disease group. In this review, ongoing ADC trials in BTCs will be discussed, with a focus on their potential role in the future management of patients with BTC.

## 2. Methods

In relation to ADCs, studies regarding use of antibody–drug conjugates in cancer, particularly in BTCs, were searched in Embase (1974 to 2024) and Ovid MEDLINE(R) (1946 to 2024). Search terms included “BTC”, “antibody drug conjugate”, “immunoconjugate”, “biliary tract neoplasms”, “tumour”, “cholangiocarcinoma”, “pancreatic cancer”, “hepatocellular cancer”, and “Trastuzumab Deruxtecan” with relevant results displayed. Meta-analyses, conference abstracts, prospective studies, and retrospective series were included. ADC use in haematological malignancies and non-English language articles were excluded. The references of eligible studies and relevant review articles (identified from the database search) were examined to detect other studies of interest. Clinical trial data were extracted from the ClinicalTrials.gov trial registry (data last accessed 5 June 2024). The summary findings are discussed in this review. 

## 3. Current Standard of Care Treatments in BTC

For patients with locally advanced or metastatic BTC, Cisplatin and Gemcitabine (CisGem) became the standard of care following the publication of the ABC-02 trial, which demonstrated a median OS of 11.7 months for the combination [17]. This was statistically significantly superior to gemcitabine alone, which yielded a median OS of 8.1 months (hazard ratio, 0.64; 95% confidence interval, 0.52 to 0.80; *p* < 0.001). More recently, the Keynote 966 and TOPAZ-1 clinical trials supported the addition of an immune checkpoint inhibitor (ICI) to CisGem in the first-line advanced setting [19,20,21]. In the Keynote 966 trial, patients with previously untreated, unresectable, locally advanced, or metastatic biliary tract cancer were randomly assigned to treatment with pembrolizumab plus gemcitabine and cisplatin, or placebo plus gemcitabine and cisplatin. OS was 12.7 months (hazard ratio, 0.83; 95% confidence interval, 11.5 to 13.6) in the pembrolizumab group versus 10.9 months (hazard ratio, 0.75; 95% confidence interval, 6.70 to 7.40) in the placebo group. Therefore, this study highlighted a meaningful improvement in OS outcomes with use of the ICI, pembrolizumab, in combination with cisplatin and gemcitabine, versus treatment with cisplatin and gemcitabine alone. In the TOPAZ-1 trial, patients with a locally advanced or metastatic BTC receiving first-line CisGem combined with the programmed cell death 1 ligand 1 (PD-L1) inhibitor durvalumab achieved a progression-free survival (PFS) of 7.2 months (hazard ratio, 0.75; 95% confidence interval, 6.70 to 7.40; *p* = 0.001) and an OS of 12.8 months (hazard ratio, 0.80; 95% confidence interval, 11.1 to 14.0; *p* = 0.021). In comparison, the arm with CisGem and the placebo achieved a PFS of 5.7 months (hazard ratio, 0.75; 95% confidence interval, 5.6 to 6.7; *p* = 0.001) and an OS of 11.5 months (hazard ratio, 0.80; 95% confidence interval, 10.1 to 12.5; *p* = 0.021). 

Following progression on first-line treatment with CisGem and durvalumab or pembrolizumab (or CisGem or Gem alone if immunotherapy is contraindicated), second-line treatment options remain limited. The current standard of care options include chemotherapy with 5-Fluorouracil (5-FU) and Oxaliplatin (FOLFOX) as in the ABC-06 trial, or 5-FU and Irinotecan (FOLFIRI) or 5-FU/liposomal irinotecan [19,20,21]. The ABC-06 trial was a randomised phase III, multicentre, controlled, open-label trial comparing FOLFOX and active symptom control (ASC) to ASC alone in patients with advanced BTC who had progressed on prior CisGem chemotherapy [22]. This trial met its primary endpoint, showing a median OS of 6.2 months in the FOLFOX and ASC arm (hazard ratio, 0.69; 95% confidence interval, 5.4–7.6; *p* = 0.031) compared to 5.3 months in the ASC alone arm (hazard ratio, 0.80; 95% confidence interval, 0.50–0.97; *p* = 0.021). In the FOLFOX and ASC arms, 69% of patients reported adverse events versus 52% in the ASC group alone. However, FOLFOX and ASC did not show a significant decline in quality-of-life parameters (quality of life and value of health questionnaires) [23]. Overall, this trial highlighted the potential benefit of FOLFOX chemotherapy in OS rates at 6 and 12 months, establishing this chemotherapy regimen as a potential standard of care option in the second-line setting in patients not eligible for targeted therapies. 

Only a small proportion of patients with BTCs are eligible for second-line treatment, with one retrospective study indicating this figure to be as low as 25% [24]. This cohort typically comprises younger patients or those with a longer PFS following first-line treatment [24]. 

## 4. Tumour Profiling and Application in BTCs

There is a pressing need for more effective and better tolerated second-line treatment options for patients with BTCs. Targeted molecular therapies are being increasingly studied and utilised for the treatment of various cancers, including BTCs, due to advances in tumour genomic profiling. Next-generation sequencing (NGS) has paved the way for identifying targetable tumour genomic alterations and molecular pathways involved in carcinogenesis [12,25]. 

The MOSCATO 01 trial utilised NGS on tumour biopsies from patients with incurable, relapsed, or resistant solid malignancies, revealing that 411 of the 843 patients had actionable molecular alterations [12]. Among these, 43 patients had advanced BTC, and 23 of them received targeted therapy. The overall response rate in this cohort was 33%, with a disease control rate as high as 88%. Notably, one patient with HER2-positive BTC, treated with a combination of trastuzumab and chemotherapy, achieved a complete response. These findings underscore the importance of molecular profiling of BTC samples following diagnosis. This approach can potentially help identify further genomic alterations that can inform additional treatment options for patients with BTCs. 

**Figure 1:**
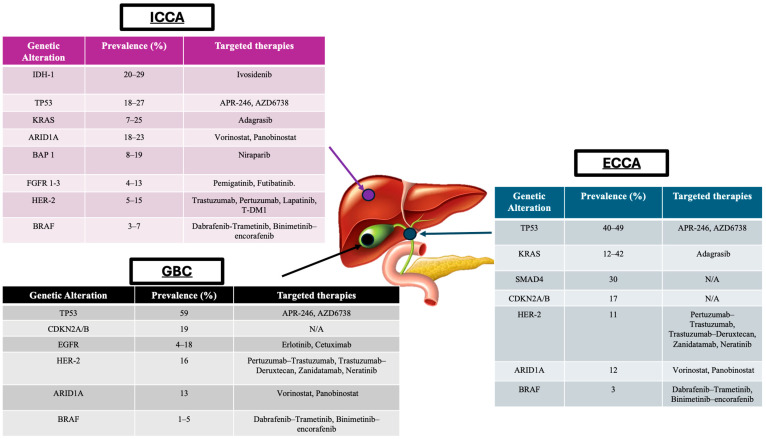
Diagram highlighting prevalence of common molecular alterations following genomic sequencing in BTC based on anatomical location and potential targeted therapies. Abbreviations: iCCA, intrahepatic cholangiocarcinoma; GBC, gallbladder cancer; eCCA; extrahepatic cholangiocarcinoma [12,29,30,31,32,33].

The prevalence of these genomic mutations vary based on the anatomical location of the tumour, highlighted by Figure 1 [13]. Thus, integrating molecular profiling into the clinical management of BTC can potentially improve outcomes for patients by allowing for more personalised approaches. Alterations found in iCCA include fibroblast growth factor receptor (*FGFR*) 1–3 and isocitrate dehydrogenase (*IDH*) mutations. *FGFR* alterations are present in up to 13% of iCCA cases [13]. Pemigatinib, an oral pan-FGFR inhibitor, is approved by the European Medicines Agency (EMA) and United States Food and Drug Administration (FDA) for patients with advanced CCA harbouring *FGFR2* fusions or rearrangement [8,26]. The FIGHT-202 trial was a multicentre, open-label, single-arm, and multicohort phase II study that recruited patients with inoperable, locally advanced, or metastatic CCA who had already progressed on at least one previous treatment. Patients with and without *FGFR* alterations received pemigatinib [27]. In the cohort with *FGFR2* rearrangements or fusions, the overall response rate (ORR) was 36.6% (95% confidence interval, 26.8–47.2), and the median PFS was 7.0 months (95% confidence interval, 6.1–10.5). Furthermore, the median OS was 17.5 months (95% confidence interval, 14.4–22.9). The FIGHT-202 study showed that pemigatinib is effective in this selected cohort of patients and is associated with manageable adverse events. However, it is important to note that acquired resistance remains a significant barrier to the efficacy of adenosine triphosphate-competitive FGFR inhibitors, such as pemigatinib, ultimately limiting their long-term efficacy. Integrating molecular profiling into the clinical management of BTCs is crucial for identifying patients who may benefit from targeted treatments. 

Isocitrate Dehydrogenase-1 (*IDH1*) mutations have been identified in an estimated 13% of iCCAs [14]. Following the positive results of the phase III ClarIDHy, the IDH1 inhibitor ivosidenib has been approved by both the EMA and FDA for patients with *IDH1* mutant locally advanced or metastatic iCCA who have been treated with at least one prior line of systemic therapy [9,10,11]. The trial met its primary endpoint of improved median PFS from 1.4 months with the placebo to 2.7 months with ivosidenib (hazard ratio 0.37, 95% confidence interval 0.25–0.54; *p* = 0.001). Updated survival data from the ClarIDHy trial shows a median OS of 10.3 months with ivosidenib (95% confidence interval, 7.8–12.4 months) versus 7.5 months with the placebo (95% CI, 4.8–11.1 months) [28]. 

In conclusion, molecular profiling has the potential to inform more personalised therapies that can improve the outcomes of patients with BTCs. However, ongoing research focuses on overcoming the challenges of acquired resistance and expanding the arsenal of targeted therapies available for patients with BTC. 

## 5. Key Components and Mechanism of Action of ADCs

**Figure 2:**
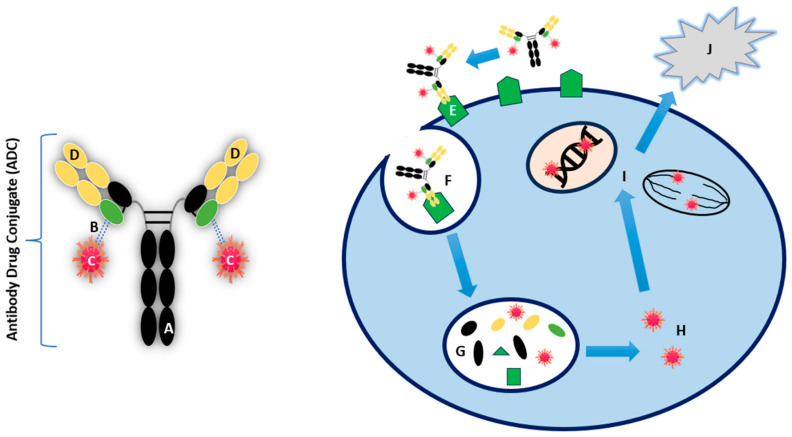
Structure and mechanism of action of ADC. (A) Humanised monoclonal antibody (mAb), (B) Linker at conjugation site (green), (C) Cytotoxic payload, (D) Antigen-binding sites (E) Target binding to cell-surface antigen, (F) Internalisation of ADC–antigen complex, (G) Lysosomal deconjugation, (H) Active cytotoxic payload release, (I) DNA/microtubule disruption, (J) Cell death [36,48,49].

Monoclonal antibodies (mAbs) have been a revolutionary part of anti-cancer therapy in recent times by directly targeting antigens that are overexpressed or selectively expressed on tumour cells [34]. More specifically, antibody-dependent cellular cytotoxicity, complement-dependent cellular cytotoxicity, and anti-angiogenic effects on tumour cells are the mechanisms that drive their efficacy. However, despite the number of mechanisms, they lack sufficient cytotoxicity. ADCs consist of three essential components: an antibody, linker, and payload, each with a vital role in ultimately causing cell death in cancer cells. The antibody controls immunogenicity and target specificity by expressing selective affinity on the target cell receptor. The linker is the structure that binds the antibody to the payload. The payload is the cytotoxic component that internalises within its target cancer cell, inducing apoptosis [35,36]. The structure and mechanism of action of ADCs is summarised in Figure 2.

The selectivity of ADCs is dictated by the antibody that recognises and targets tumour cells overexpressing a specific antigen, thereby sparing normal human tissue. For example, the ADC Trastuzumab emtansine (T-DM1) consists of trastuzumab, an antibody targeting tumour cells overexpressing the human epidermal growth factor 2 (HER2), linked with the antimitotic agent emtansine (DM1). HER2 is overexpressed in about 20% of breast cancers, where T-DM1 allows delivery of the payload specifically to these cancer cells alone, resulting in minimal toxicity to non-malignant tissue and improving the therapeutic index [37,38]. T-DM1 has been approved by National Institute for Health and Care Excellence (NICE) since 2017 for treating HER2-positive, unresectable, locally advanced, or metastatic breast cancer in adults who previously received trastuzumab or taxane [39]. Targeting HER2 in BTC will be discussed later in this review. 

The linker is pivotal in ensuring that the antibody and payload are bound whilst in the bloodstream, subsequently releasing the cytotoxic payload into the target cancer cells. Therefore, it is the component that determines the stability of an ADC, its pharmacokinetics, and pharmacodynamics. Linkers can either be cleavable or non-cleavable. Cleavable linkers can be degraded in certain environments, for example, based on pH levels, enzymatic activity (proteolysis), or glutathione levels [40]. This can be via endosomes or lysosomes. In contrast, non-cleavable linkers require complete lysosomal degradation for the payload to be released. They have shown superior safety profile than ADCs with cleavable linkers, owing to their increased stability in the circulation and a longer half-life [40,41].

The payload represents the final key component of the ADC by delivering the chemotherapeutic agent to the tumour cell following internalisation. These payloads include tubulin inhibitors, immunomodulators, and deoxyribonucleic acid (DNA)-damaging agents [36,40,41]. ADCs primarily target positive or neutral charges on the tumour cells for selective cytotoxicity. Specific charges on the payload and linker are carefully engineered to influence targeting, delivery, and solubility [42]. Positively charged payloads tend to interact more effectively with the negatively charged phospholipid membranes of cancer cells, aiding in cellular uptake and internalisation. One example is the use of monomethyl auristatin E (MMAE), a commonly used payload that is positively charged at physiological pH and has demonstrated strong cytotoxic activity in targeted cancer cells [36,43]. However, balancing the charge is vital, as highly charged molecules can result in off-target toxicity or rapid systemic clearance. Neutral or hydrophilic payloads are often favoured as they reduce off-target toxicity by limiting nonspecific interactions with other cells or proteins in the bloodstream. They can reduce aggregation of the ADC in the circulation and prevent rapid clearance or immune system activation [44]. Neutral payloads passively diffuse across the cell membrane, contributing to the so-called “bystander effect,” where the drug affects both targeted and neighbouring cells, enhancing efficacy in heterogeneous tumours [45]. The selection of the payload in ADCs is driven by several factors, including potency, mechanism of action, stability, and specificity to cancer cells [46,47].

## 6. Application of Biomarkers during ADC Treatment

A biomarker, in the context of cancer, represents a biological molecule that can be found in blood, other bodily fluids, or tissue associated with a specific biological process such as tumour development or response to therapy. These alterations may be due to germline or somatic mutations, transcriptional changes, epigenetic signatures, etc. [50]. Various types of biomarkers include proteins (enzyme or receptor), nucleic acids, antibodies, and peptides [51]. Diagnostic biomarkers can help detect the presence of cancerous cells, such as prostate-specific antigen (PSA) in prostate cancer [52,53]. Prognostic biomarkers can help outline the likely course or outcome of the disease and guide how aggressive the cancer may behave, e.g., *BRCA* 1/2 mutations in assessing breast and ovarian cancer risk [54,55,56]. Predictive biomarkers can help indicate how likely a patient will respond to a particular treatment, helping to guide personalised therapy. For example, HER-2 overexpression in breast cancer predicts response to HER-2 targeted therapies (e.g., Trastuzumab) [57,58].

A good biomarker for ADCs should possess certain characteristics that allow the ADC to effectively target and kill the cancer cells. These biomarkers help guide the selection of patients who are most likely to benefit from ADC therapy and improve the precision and efficacy of treatment. These characteristics include (1) high and stable expression in tumour cells; ensuring that the ADC can reliably bind to the target, as a low or variable expression, may result in suboptimal delivery of the cytotoxic payload [40,59]. Furthermore, tumour-specific antigens that are minimally expressed have been shown to reduce the effects of off-target toxicities. (2) The ability to internalise upon ADC binding is needed; a good biomarker must trigger internalisation after the ADC binds to it to ensure the ADC–antigen complex enters the cancer cell and releases the cytotoxic drug [36]. (3) Functionally relevant to cancer biology, biomarkers play a key role in tumour growth, survival, and progression and therefore are targeted to deliver the cytotoxic payload. It can also contribute to disrupting cancer pathways. For example, the HER-2-propagated, PI3K/Akt/mTOR signalling pathway enhances cancer survival and migration in the metastatic microenvironment, initiating secondary tumours [60]. Therefore, the ADC, T-Dxd, aims at inhibiting HER-2 signalling and slowing the growth of the cancer cells. This has proven to be an effective targeted treatment, such as in breast cancer, which will be highlighted later in this review. (4) For the accessibility to testing, the biomarker must be measurable through clinical testing (e.g., immunohistochemistry (IHC) or genetic profiling), which can help identify patients who are suitable to receive ADC therapy and are most likely to benefit from treatment [61,62]. For example, in breast cancer, T-DM1 relies on a high expression of HER-2 (*HER2* gene amplification or IHC +3 on staining) for efficacy as it relies on HER2 signalling for optimal binding and internalisation. In contrast, T-Dxd is less reliant on HER-2 expression as it has shown efficacy in HER-2-low cancers as well (*HER2* gene non-amplified or IHC +1 or +2 on staining) [63,64]. This can be attributed to its more potent payload (drug–antibody ratio 8:1) and “bystander effect”, which will be outlined later in this review [65]. 

Biomarkers are crucial for early detection, personalised treatment, and the monitoring of cancer progression or treatment response. 

## 7. Advancements in ADC Research and Development

Paul Ehrlich, a German scientist, laid the foundation for the concept of antibody–drug conjugates (ADCs) through his “magic bullet” theory [66]. He proposed that antibodies could be used to selectively target disease-causing pathogens without harming the host, an idea that later became crucial for ADC development. The use of monoclonal mAbs to target tumour antigens advanced after the development of hybridoma technology in 1975, enabling the production of mAbs in the lab [67].

In the 1990s, preclinical studies highlighted the potential of ADCs by improving the therapeutic index of cytotoxic drugs and enhancing their selectivity for tumour cells [68,69]. This led to the approval of gemtuzumab ozogamicin in 2000, the first FDA-approved ADC for treating acute myeloid leukaemia (AML), which paved the way for further ADC research [70].

Modern advancements in ADCs focus on identifying new antigen targets or antibodies, improving linker stability and design, optimising pharmacokinetics, expanding the use of different cytotoxic payloads, and overcoming resistance mechanisms [36,71].

The FDA approval of trastuzumab deruxtecan (T-DXd) marked a milestone for ADCs in biliary tract cancer (BTC) [72]. T-DXd uses an anti-HER2 monoclonal antibody linked to a topoisomerase I inhibitor, deruxtecan (Dxd), via a cleavable peptide linker [73,74]. Dxd is a highly potent payload, reported to have 10-fold higher potency than SN-38, the active metabolite of irinotecan [75,76]. Its high drug–antibody ratio (DAR) of 8 enhances its effectiveness in targeting HER2-positive BTC, and its stable linker reduces systemic toxicity [73].

While T-DXd is the only ADC approved for BTC so far, ongoing preclinical studies are exploring other antigens and ADCs in the hopes of expanding targeted treatment options in this cancer type [77].

## 8. ADCs in Breast Cancer

ADCs are an emerging targeted therapeutic strategy that show promising efficacy and safety and may offer a solution to tumour resistance in many solid tumour types, including BTCs. Some examples of their use in various solid tumours will now be presented. 

In August 2022, the United States Food and Drug Administration (FDA) approved T-Dxd for the treatment of patients with unresectable or advanced HER2-positive breast cancer who have previously been treated with an anti-HER2-based regimen and have developed disease recurrence during or within 6 months of completing therapy [78,79]. This approval followed the results of the DESTINY-Breast 03 trial, an open-label, randomised, and multicentre phase III trial that compared T-Dxd to T-DM1 [78]. T-Dxd demonstrated a statistically significant improvement in ORR [79% (95% confidence interval, 73.1–83.4) versus 35% (95% confidence interval, 29.2–41.1)]. T-Dxd also led to a superior median PFS [29 months (95% confidence interval, 23.7–40.0) versus 7.2 months (95% confidence interval, 6.8–8.3)] and median OS [52.6 months (95% confidence interval, 48.7-non estimable) versus 42.7 months (95% confidence interval, 35.4-non estimable)]. Moreover, it had a manageable safety profile, reflected in the superior treatment duration compared to T-DM1. 

In contrast to T-DM1, T-Dxd has proven effective even in HER-2-low (IHC +1 or IHC +2/in situ hybridisation negative)-expressing metastatic breast cancer. It has been approved by the FDA, following outcomes of the DESTINY-Breast 04 trial, where T-Dxd highlighted improved PFS [9.9 months (95% confidence interval, 9.0–11.3) versus 5.1 months (95% confidence interval, 4.2–6.8)] and OS [23.4 months (95% confidence interval, 20.0–24.8) versus 16.8 months (95% confidence interval, 14.5–20.0)] in patients with HER-2-low-expressing metastatic breast cancer, compared to treatment of physician’s choice (capecitabine, eribulin, gemcitabine, paclitaxel, or nab-paclitaxel) [65]. This cohort of patients were traditionally not considered eligible for HER2-targeted therapies, but T-DXd’s ability to kill cancer cells through its potent cytotoxic payload and bystander effect (where the drug affects neighbouring cancer cells regardless of HER2 status) has led to significant clinical responses. This is particularly important in tumours with heterogeneous HER2 expression, expanding the range of cancers that can potentially be treated with this ADC. 

## 9. ADCs in Lung Cancer

Additionally, T-Dxd was the first ADC approved for use in patients with HER2-mutant non-small cell lung cancer (NSCLC) following the results of the DESTINY-Lung01 and DESTINY-Lung02 trials, reporting an ORR of 56% (95% confidence interval, 41.73–70.0) and a duration of response (DOR) of 16.8 months (95% confidence interval, 6.4-not estimable) [80,81]. The estimated median PFS was 8.2 months, and the median OS was 18.6 months. While the study concluded that T-Dxd had an overall acceptable safety profile at a dosage of 5.4 mg/kg (as opposed to 6.4 mg/kg), it is worth noting that rates of treatment-related interstitial lung disease (ILD) were up to 12.9% in the 5.4 mg/kg cohort and 28% in the 6.4 mg/kg cohort. In the aforementioned DESTINY-Breast03 trial, the most common treatment-related adverse events (TRAEs) that led to discontinuation were pneumonitis (15 out of 51 patients) and ILD (13 out of 51 patients). Among patients with metastatic breast cancer and HER2-mutant NSCLC treated with T-Dxd, fatal outcomes due to ILD or pneumonitis were reported in 1% of the cohort [82]. Therefore, close monitoring and the early recognition of potential respiratory symptoms are essential, particularly in anti-HER2-based ADCs. A pooled analysis of phase I and II T-Dxd monotherapy studies by Powell et al., which included 1150 patients of multiple tumour types (including breast, gastric, colorectal and lung cancer), concluded that adjudicated drug-related ILD/pneumonitis occurred in 15.4% of patients, most commonly within the first year of treatment [83]. Practical strategies to tackle ADC treatment-related ILD, as evidenced by Rugo et al., include pre-treatment patient screening, recognising appropriate diagnostic imaging (high-resolution CT), multidisciplinary input, patient education, knowing when to suspend treatment, and appropriate steroid dosing based on the toxicity grade [84]. 

These findings highlight the need for vigilant monitoring and management strategies to mitigate the risk of ILD in patients undergoing treatment with ADCs. T-Dxd, in particular, has been highlighted as an agent associated with higher prevalence of treatment-related ILD in comparison to other ADCs [85]. Therefore, implementing these strategies may help improve patient outcomes and ensure the safe use of these promising targeted therapies. 

## 10. ADCs in Urothelial Cancer

In recent times, ADCs have also emerged as a promising therapeutic approach for urinary tumours. Their role in prostate and renal cancers remains in the early stages of investigation, but they are currently explored in larger studies. In urinary tumours, ADCs can potentially provide a safer alternative to current standard of care systemic chemotherapy regimens for patients with compromised renal function due to their ability to minimise off-target effects [86]. The targets of ADCs for urothelial carcinoma are mainly three membrane proteins: HER-2, TROP2, and Nectin-4 [87]. 

Practice changing enfortumab vedotin (EV) plus pembrolizumab was investigated in the phase III EV-302 trial, which randomised 886 patients with previously untreated locally advanced or metastatic urothelial carcinoma to either EV plus pembrolizumab or gemcitabine in combination with either cisplatin or carboplatin [88]. After a median follow-up of 17.2 months, the median PFS was significantly longer with EV plus pembrolizumab compared to chemotherapy (12.5 months vs. 6.3 months; HR, 0.45; 95% CI, 0.38–0.54; *p* < 0.001). The median OS was also significantly longer with EV plus pembrolizumab (31.5 months vs. 16.1 months; HR, 0.47; 95% CI, 0.38–0.58; <0.001). Confirmed ORR was 67.7% and 44.4% for EV plus pembrolizumab and chemotherapy, respectively (*p* < 0.001), with complete responses (CRs) observed in 29.1% of patients in the EV plus pembrolizumab group. 

Sacituzumab govitecan is another antibody–drug conjugate composed of an anti-Trop-2 humanised monoclonal antibody coupled to SN-38, the active metabolite of the topoisomerase 1 inhibitor irinotecan [89]. At a median follow-up of 9.1 months, ORR was 27% (95% CI, 19.5%–36.6%) and 77% of participants showed a decrease in measurable disease (in patients with locally advanced unresectable or metastatic urothelial cancer). The median DOR was 7.2 months (95% CI, 4.7–8.6 months), median PFS was 5.4 months (95% CI, 3.5–7.2 months), and median OS was 10.9 months (95% CI, 9.0–13.8 months) [89]. T-Dxd data were also reviewed for patients with bladder cancer as part of DESTINY-Pan-tumour02 trial [90]. 

## 11. Use of T-Dxd in a Pan-Tumour Trial

As evidenced in Figure 1, *HER2* mutations can be found in up to 15% of patients with iCCA, 16% of those with GBC, and 11% of those with eCCA. Given the promising outcomes following the use of ADCs in breast and lung cancer, these therapies are now being investigated in other tumour types harbouring *HER2* alterations. 

It is worth noting the differences between HER2 overexpression (or amplification) and *HER2* mutations, particularly in the context of treatment with ADCs. HER2 overexpression refers to excess production of the HER2 protein on the cell surface due to gene amplification, thereby resulting in an abnormally high number of HER2 receptors on the surface of the cancer cells [91,92]. Therefore, ADCs such as T-Dxd utilise their antibody component to specifically target these cells with high HER2 expression allowing internalisation and delivery of the cytotoxic payload. HER2 overexpression can be identified through methods such as IHC, which measures the protein overexpression or fluorescence in situ hybridisation (FISH), which measures the number of HER2 gene copies in the cancer cells [92,93]. 

*HER2* mutation refers to specific changes in the DNA sequence of the *HER2* gene that can lead to a permanent activation of the HER2 protein, even without gene amplification. Although these mutations may not cause overexpression of the protein, they are still a driving factor for signalling pathways associated with carcinogenesis [94]. ADCs may be effective in this context if there is a high enough level of the mutant HER2 protein expressed on the cell surface that the antibody component can target. Furthermore, some mutations can affect the binding affinity of the ADC, which can diminish their efficacy [43]. Therefore, ADC treatment in cancers with *HER2* mutations are more variable and depend on the specific nature of the mutation. *HER2* mutations can be identified through methods such as next-generation sequencing (NGS), which can detect specific mutations in the sequence of the *HER2* gene (point mutations, deletions, etc.) [95,96]. It can also be identified through use of polymerase chain reaction (PCR)-based assays that target known mutations in the *HER2* gene [95]. 

The DESTINY-PanTumor02 trial was an open-label, multicentre, phase II study that assessed the efficacy and safety profile of T-Dxd in HER2-overexpressing solid tumours. The patients recruited included those with histologically confirmed locally advanced or metastatic solid HER2-overexpressing (immunohistochemistry +2/+3) tumours across seven different disease groups, including BTCs (*n* = 41), that had progressed or were refractory to alternative systemic treatment options (including prior HER2 therapy). Of the 267 patients included, 99 achieved tumour response, corresponding to an ORR of 37%. In the BTC cohort, the ORR was 22% (95% confidence interval, 10.6–37.6). Notably, those with HER2 IHC +3 expression across all disease groups achieved the longest DOR, PFS, and OS, with an ORR of 56.3% and a median OS of 12.4 months in the BTC cohort [90]. Another phase II trial also analysed the use of T-Dxd in HER2-expressing BTCs, suggesting this may be a promising targeted therapy for the future [97]. 

A phase I study presented at the American Society of Clinical Oncology (ASCO) meeting in 2024 showed promising preliminary results for the ADC IBI343 in patients with Claudin 18.2-expressing advanced pancreatic ductal adenocarcinoma (PDAC) and BTCs that were intolerant or refractory to standard treatments [98]. In this study, twenty-eight patients with PDAC and seven patients with BTCs were included. Seven patients achieved a partial response (PDAC *n* = 5, BTC *n* = 2), with an ORR of 38.5% (95% confidence interval, 13.9–68.4) and a disease control rate (DCR) of 84.6% (95% confidence interval, 54.6–98.1). Treatment-related adverse events (TRAEs) occurred in 80% of patients, although only 25% experienced grade 3 or higher TRAEs. Overall, this study highlighted promising efficacy and tolerability profiles for IBI343, prompting further larger population studies.

ADCs are associated with several potential side effects, including haematological toxicities, nausea, vomiting, diarrhoea, and less commonly ocular toxicities or ILD [99]. The side effects caused by ADCs can be explained by different mechanisms, including the type of payload (hydrophobic versus hydrophilic), type of linker (cleavable versus non-cleavable), and drug-to-antibody ratio (DAR). The “bystander effect” is a vital component of ADCs in bypassing tumour heterogeneity, but it is complicated by releasing systemic effects to non-target cells that carry the target antigen (on-target, off-tumour toxicity) [99,100]. Hydrophobic payloads with strong membrane penetration result in a potent bystander effect; however, this also means the drug is easily absorbed by healthy tissue, resulting in systemic side effects [99]. Further investigation is required to ascertain the correct combination of the antibody target, drug-linker, DAR, and dosing regimens to improve the safety profiles of ADCs. 

## 12. Current Trials Assessing the Use of ADCs in BTCs 

Ongoing phase I and II clinical trials are currently recruiting patients with cancers, including BTCs, aiming to assess the efficacy and safety of treatment with ADCs in this cohort and identifying further actionable tumour alterations (Table 1). For example, MRG003, is an ADC comprised of an anti-epidermal growth factor receptor (EGFR) humanised immunoglobulin G1 monoclonal antibody that is conjugated with monomethyl auristatin E via a valine-citrulline linker. As evidenced in Table 1, this ADC is being utilised in an open-label, single-arm, and multicentre phase II clinical study on the treatment of *EGFR*-positive unresectable, locally advanced, or metastatic BTC that have progressed during or relapsed after at least one previous standard therapy (NCT04838964). The projected enrolment for this cohort is 80 patients. *EGFR* somatic mutations can be present in 4–18% of GBCs, as highlighted in Figure 1, whilst EGFR amplifications are associated with poorer prognosis and occur in 19–31% of iCCAs or eCCAs [101]. MRG003 has already shown encouraging outcomes, following results of another phase I trial, when used in the treatment of patients with *EGFR* positive refractory advanced squamous cell carcinomas of the head and neck (SCCHN), nasopharyngeal carcinoma (NPC), and colorectal cancer (CRC) [102]. The confirmed ORR was 40% in the cohort with SCCHN and 44% with NPC. Although a small sample size (*n* = 61), the promising anti-tumour effects highlighted in this study and the acceptable safety profile are promising, and it is hoped that these outcomes can be replicated in the BTC trial (NCT0483894). 

AZD8205 is another ADC comprised of human anti-B7-H4 antibody conjugated via a chemical linker to a topoisomerase I inhibitor payload. As evidenced in Table 1, it is being investigated in an open-label, multicentre phase I/IIa trial as a monotherapy or in combination with other anti-cancer agents in patients with advanced or metastatic solid tumours, including breast, endometrial, ovarian, and BTCs (NCT051203482). The estimated enrolment for this study is 340 patients. B7-H4 is overexpressed in multiple tumour types and is known to negatively regulate the T cell-mediated anti-tumour immune response [103,104]. The analysis of tumour samples using Western blotting and quantitative real-time PCR in patients with iCCA highlighted higher levels of B7-H4 expression compared to peri-tumoural tissues. Furthermore, a high B7-H4 expression in these tumour samples were associated with lymph node metastasis, advanced tumour stage, and poorer differentiation, with shorter OS and disease-free survival in this cohort of patients [103]. Therefore, this may be an additional targetable tumour alteration in BTC (NCT051203482). 

Datopotamab deruxtecan (Dato-Dxd) is an ADC comprised of a trophoblast cell surface antigen 2 (TROP2)-directed monoclonal antibody linked to the topoisomerase I inhibitor payload deruxtecan via a cleavable linker. As evidenced in Table 1, it is being investigated in an open-label, multicentre phase II trial as a monotherapy or in combination with other anti-cancer agents in patients with advanced or metastatic solid tumours, including BTCs, colorectal, endometrial, gastric, ovarian, prostate, and urothelial cancers (NCT05849211). The estimated enrolment for this study is 582 patients. TROP2 overexpression in GBC tumour samples has been associated with the promotion of cell proliferation, clone formation, invasion, and migration in vitro through the regulation of the phosphoinositide-3-kinase–protein kinase B/Akt (PI3K-PKB/Akt) pathway [105]. Dato-Dxd has a DAR of 4, allowing for a wider therapeutic window [106]. It has resulted in encouraging outcomes in the open-label, randomised, phase III TROPION-lung01 trial in patients with advanced non-small cell lung cancer with an actionable genetic alteration, assigned to treatment with Dato-Dxd or docetaxel (DTX) [107]. Patients treated with Dato-Dxd achieved a superior ORR (26.4% versus 12.8%) and treatment duration [4.2 months (range 0.7–18.3 months) versus 2.8 months (range 0.7–18.9 months)] when compared to DTX. 

It is uncertain if other molecular alterations will be investigated as potential targets for ADC development in the future. As highlighted in Figure 1, other alterations such as *TP53*, *KRAS*, and *ARID1A*, which are more commonly associated with BTC tumours, may be targets of interest in future ADC development. *TP53* and *KRAS* are well-known oncogenes, and their mutations are common in aggressive cancers like BTC. *KRAS* mutations are found in approximately 90% of patients with metastatic PDAC, specifically *KRAS G12D* and *KRAS G12V* comprising the majority of these identified mutations [108]. KRAS G12D directly drives the expression of the glycoprotein, intracellular adhesion molecule 1 (ICAM1), which is abnormally overexpressed in a variety of cancers including PDACs [109,110]. One particular preclinical study has shown promising results in targeting this pathway by utilising an ADC called ICAM1-DM1. The use of this ADC demonstrated significant regression in tumour growth and metastasis when examined in an orthotopic pancreatic cancer model [111]. Future trials may explore *ARID1A* mutations as a target, which are involved in chromatin remodelling, and are increasingly recognised in BTCs, especially intrahepatic cholangiocarcinoma. 

It is hoped that outcome data from the ongoing trials utilising ADCs in the treatment of patients with BTC may help identify key components that drive the efficacy, e.g., the charge of the payload (discussed in Section 5 of this review). T-Dxd uses a non-charged topoisomerase I inhibitor as the payload, allowing it to be more effective in HER2-low and heterogeneous cancer cells. Future ADC development could focus on identifying other suitable payload charges. DM1 and DM4 are derivatives of the cytotoxic agent maytansine used in ADCs like T-DM1. These payloads are neutral, which helps improve their therapeutic index by minimising off-target binding and immune clearance, while maintaining potent cytotoxic activity via microtubule disruption. Calicheamicin, used in gemtuzumab ozogamicin (Mylotarg) for acute myeloid leukaemia (AML), is another example of a neutral payload. It binds to DNA and induces double-strand breaks, leading to apoptosis. Its neutral charge aids in targeted delivery and avoids rapid clearance from circulation [112].

Notably, these select trials highlighted in Table 1 involve the treatment of patients with BTC in the locally advanced or metastatic setting; therefore, depending on the outcomes from these advanced trials, future trials may investigate the use of ADCs in the treatment of patients with BTC in the neoadjuvant or adjuvant setting. Most ongoing trials, as noted in Table 1, are primarily evaluating pharmacokinetics, safety, and efficacy as their primary endpoints. Pharmacokinetic studies help in understanding how the drug is absorbed, distributed, metabolised, and excreted, while safety profiles and efficacy data will establish the therapeutic window and benefit–risk ratio of each ADC in BTC patients. Currently, T-DXd is the only ADC approved by the FDA for the treatment of unresectable or metastatic HER2-positive BTC. The paucity of outcome and safety data in BTC emphasises the need for more studies to expand the clinical application of ADCs in this disease group. The trials under review are expected to provide important data on the efficacy, safety, and overcoming issues with resistance, potentially broadening the use of ADCs in BTC. Expanding the data on ADCs could lead to their wider adoption in BTC, not only for HER2-positive disease but possibly for other subtypes, depending on the molecular landscape and drug development.

## 13. Limitations and Ongoing Challenges Associated with ADCs

Despite their emergence as a useful drug class in oncology, the application of ADCs in clinical practice faces several limitations, necessitating the creation of an ideal agent that balances optimum efficacy with minimal toxicity. 

The key challenges are as follows: (1) Overcoming off-target effects: By identifying target antigens that are highly specific to cancer cells, off-target binding can lead to the damage of healthy tissues and adverse side effects. Research is ongoing to find biomarkers that are exclusively or predominantly expressed on cancer cells [42,113]. (2) Minimising ADC immunogenicity: Immunogenic responses can reduce the efficacy of ADCs and cause unwanted immune reactions. Strategies to reduce immunogenicity include using humanised or fully human antibodies and modifying linker and payload components to reduce recognition by the immune system [42,113]. (3) Stability of cytotoxic payload: Ensuring that the cytotoxic payload remains stable during conjugation and circulation is essential. The premature release of the payload can reduce therapeutic efficacy and increase toxicity. Developing stable linker technologies that only release the payload in the target environment (e.g., the tumour microenvironment) is a key focus area [63,71]. (4) Optimising pharmacokinetic properties: Tailoring the pharmacokinetic properties of ADCs to achieve the necessary systemic clearance and optimal tumour penetration is vital. This involves adjusting the size, charge, and other properties of the ADC to enhance its ability to reach and be retained in the tumour tissue while avoiding rapid clearance from the bloodstream [36,43,44]. (5) Cost-effectiveness: Developing cost-effective ADCs is crucial for their widespread adoption and accessibility. The manufacturing process of ADCs is complex and expensive, involving the production of antibodies, cytotoxic drugs, and linkers, and the conjugation process itself is complex. Streamlining production processes and improving efficiency may help reduce costs [42,71]. (6) Acquired resistance: This issue remains an emerging challenge in ADCs, as is the case with many standard chemotherapies, where tumours develop mechanisms to evade their effects over time. This resistance may have arisen from the start of treatment (de novo) or following treatment with the drug (acquired). The causes underlying ADC resistance is not fully characterised as this remains a novel form of treatment with a complex mechanism of action [40]. Therefore, preclinical studies have hypothesised potential mechanisms of resistance that stem from each component of the ADC [114]. Antigen downregulation: ADCs rely on the specific expression of antigens on cancer cells; therefore, if these tumour cells lose expression of target antigens, then ADCs may no longer effectively bind and destroy the cancer cells [114,115]. Altered internalisation and trafficking: ADCs must be internalised by the cancer cells for the cytotoxic payload to be released. Changes in receptor-mediated endocytosis or intracellular trafficking can prevent the drug from reaching its intended target inside the cell [116,117]. Drug efflux mechanisms: Cancer cells can overexpress efflux transporters such as P-glycoprotein, which pump the cytotoxic payload of the ADC out of the cell, reducing its intracellular concentration and diminishing its effectiveness [60]. The overexpression of multidrug resistance-associated protein 1 (MRP1) was demonstrated in different T-DM1 cell lines [114]. Alterations in payload sensitivity: Tumour cells may develop resistance to the cytotoxic drug itself. This can occur via the upregulation of DNA repair mechanisms, changes in cell cycle regulation, or mutations that render the cell less susceptible to the drug’s effects [118]. Tumour microenvironment: The microenvironment surrounding the tumour can influence ADC efficacy. For instance, factors like hypoxia, low pH, or stromal cell interactions may affect drug release, cellular uptake, or ADC stability, potentially leading to resistance [119,120].

Addressing these challenges involves a multidisciplinary approach, combining advances in molecular biology, chemistry, pharmacology, and bioengineering [71,114].

## 14. Conclusions and Future Directions

Biliary tract cancers remain a challenging group of malignancies to treat, especially in advanced stages, due to their poor prognosis and limited treatment options. Despite the reliance on chemotherapy and immunotherapy as the primary treatment modalities in cases where patients are fit to receive systemic treatment, ADCs have emerged as a promising targeted therapy option. ADCs offer improved cytotoxicity, specifically against cancer cells, while potentially reducing systemic toxicities [37].

A notable advancement in ADCs is the development of anti-HER2 ADCs, such as T-Dxd. T-Dxd has shown significant survival benefit in patients with HER2-positive solid tumours. In recognition of these benefits, the FDA granted approval in April 2024 for the use of T-Dxd in adult patients with unresectable or metastatic HER2-positive solid tumours, who have previously undergone systemic treatment and lack satisfactory alternative treatment options [72]. In September 2023, the EMA granted approval for the use of T-Dxd as a monotherapy in adult patients with unresectable or metastatic HER2-positive breast cancer, who have received one or more prior anti-HER2-based regimens, and also for the treatment of adult patients with unresectable or metastatic HER2-low breast cancer, who have received prior chemotherapy in the metastatic setting or developed disease recurrence during or within 6 months of completing adjuvant chemotherapy. It was also approved for the treatment of adult patients with advanced NSCLC whose tumours have an activated *HER2* (*ERBB2*) mutation and who require systemic therapy following platinum-based chemotherapy with or without immunotherapy, and for adult patients with advanced HER2-positive gastric or gastro-oesophageal junction adenocarcinoma who have received a prior trastuzumab-based regimen [121]. These approvals underscore the potential for T-Dxd to improve outcomes for patients with challenging malignancies, including BTCs.

Current trials are ongoing to explore other ADC actionable alterations in BTCs, as has been highlighted in this review. However, despite their emergence as a useful drug class in oncology, there remains certain limitations, with a pressing need to create a cost-effective agent that possesses optimum efficacy with minimal toxicity. Overall, this review has highlighted that ADCs have shown encouraging outcomes in cancer therapy, and this should lead to further research including in BTCs, where treatment options are often limited. The promising results observed with ADCs in various cancers underscore their potential as a transformative approach in oncology, warranting continued exploration and development and the need for education on the management of their specific toxicities. By addressing current challenges and optimising ADC design and application, future studies could potentially improve treatment outcomes for patients with BTCs and beyond, potentially in both early and advanced stage settings.

## Figures and Tables

**Table 1:** Some current phase I and II trials assessing the efficacy and safety of ADCs in BTC, which aim to analyse survival outcomes, identify further actionable biomarkers, and highlight which patient populations would most likely gain clinical benefit.

ID	Title of Study	Antibody Drug Conjugate	Primary Site	Primary Outcome	Target Recruitment Number
NCT04482309	A Phase 2, Multicentre, Open-label Study to Evaluate the Efficacy and Safety of Trastuzumab Deruxtecan (T-DXd, DS-8201a) for the Treatment of Selected HER2 Expressing Tumours (DESTINY-PanTumor02)	Trastuzumab Deruxtecan	Biliary tract, bladder, cervical, colorectal, endometrial, epithelial, gastric, non-small cell lung, ovarian, pancreatic.	Evaluate the efficacy and safety of Trastuzumab Deruxtecan (T-DXd) for the treatment of selected HER2-expressing tumours	468
NCT04644068	A Modular Phase I/IIa, Open-label, Multicentre Study to Assess the Safety, Tolerability, Pharmacokinetics, Pharmacodynamics and Preliminary Efficacy of Ascending Doses of AZD5305 as Monotherapy and in Combination With Anti-cancer Agents in Patients With Advanced Solid Malignancies	Trastuzumab Deruxtecan	Biliary tract, ovarian, breast, pancreatic, prostate, small cell and non-small cell lung, colorectal, bladder, gastric, cervical, endometrial	Determine if experimental treatment with PARP inhibitor, AZD5305, alone, or in combination with anti-cancer agents is safe, tolerable, and has anti-cancer activity in patients with advanced solid tumours.	804
NCT04838964	An Open-label, Single-arm, Multi-center, Phase II Clinical Study of MRG003 in the Treatment of Patients With EGFR-positive Unresectable, Locally Advanced or Metastatic Biliary Tract Cancer	MRG003	Advanced or metastatic biliary cancer	Assess the safety, efficacy, pharmacokinetics, and immunogenicity of MRG003 as single agent in EGFR-positive unresectable locally advanced or metastatic biliary tract cancer patients who have progressed during or relapsed after at least one prior standard therapy.	80
NCT05123482	A Phase I/IIa Multi-center, Open-label Master Protocol Dose Escalation and Expansion Study of AZD8205 as Monotherapy and in Combination With Anticancer Agents in Participants With Advanced Solid Tumours (BLUESTAR)	AZD8205	Biliary tract, breast, endometrial, ovarian	Study a possible treatment for advanced or metastatic solid tumours alone or in combination with anti-cancer agents	340
NCT05489211	A Phase II, Multicentre, Open-label, Master Protocol to Evaluate the Efficacy and Safety of Datopotamab Deruxtecan (Dato-DXd) as Monotherapy and in Combination With Anticancer Agents in Patients With Advanced/Metastatic Solid Tumours	Datopotamab Deruxtecan	Biliary tract, urothelial, colorectal, ovarian, endometrial, gastric, prostate	Investigate the safety, tolerability, and anti-tumour activity of Datopotamab Deruxtecan (Dato-DXd) as monotherapy and in combination with anti-cancer agents in patients with advanced/metastatic solid tumours.	582
NCT04329429	An Open-label, Single-arm, Multi-center, Phase II Study of RC48-ADC in Subjects With HER2 Overexpressed Locally Advanced or Metastatic Biliary Tract Cancer (BTC) Who Have Failed First-line Chemotherapy	RC48-ADC	Biliary tract	Evaluate the efficacy and safety of intravenous RC48-ADC in patients with locally advanced or metastatic HER2 overexpressed biliary tract cancer who have failed first-line chemotherapy.	57

ClinicalTrials.gov [Last Accessed 5 June 2024].
